# Time Course and Risk Profile of Work-Related Neck Disability: A Longitudinal Latent Class Growth Analysis

**DOI:** 10.1093/ptj/pzac050

**Published:** 2022-04-28

**Authors:** Yanfei Xie, Brooke K Coombes, Lucy Thomas, Venerina Johnston

**Affiliations:** School of Health and Rehabilitation Sciences, The University of Queensland, Queensland, Australia; RECOVER Injury Research Centre, The University of Queensland, Queensland, Australia; School of Health Sciences and Social Work, Griffith University, Queensland, Australia; Menzies Health Institute Queensland, Griffith University, Queensland, Australia; RECOVER Injury Research Centre, The University of Queensland, Queensland, Australia; School of Health and Rehabilitation Sciences, The University of Queensland, Queensland, Australia; RECOVER Injury Research Centre, The University of Queensland, Queensland, Australia

**Keywords:** Biopsychosocial Risk, Central Sensitization, Disability Trajectory, Neck Pain, Prospective Study, Quantitative Sensory Testing

## Abstract

**Objective:**

Given the economic burden of work-related neck pain and disability, it is important to understand its time course and associated risk factors to direct better management strategies. This study aimed to identify the 1-year trajectories of work-related neck disability in a high-risk occupation group such as sonography and to investigate which baseline biopsychosocial factors are associated with the identified trajectories.

**Methods:**

A longitudinal study was conducted among 92 sonographers with neck disability assessed at 3 time points—baseline, 6 months, and 12 months—using the Neck Disability Index. Baseline biopsychosocial measures included individual characteristics (demographics and physical activity levels), work-related physical and psychosocial factors (eg, ergonomic risk, workplace social support, job satisfaction), general psychological features (depression, anxiety, pain catastrophizing, and fear-avoidance beliefs), and quantitative sensory testing of somatosensory function (cold and pressure pain thresholds at neck and tibialis anterior, and temporal summation).

**Results:**

Two distinct trajectories of neck disability were identified, including a “low-resolving disability” trajectory showing slow improvement toward no disability (64.8%) and a “moderate-fluctuating disability” trajectory characterized by persistent moderate disability with a small fluctuation across time (35.2%). The trajectory of moderate-fluctuating disability was associated with more severe symptoms, lower vigorous physical activity, higher ergonomic risk, remote cold hyperalgesia, widespread mechanical hyperalgesia, heightened pain facilitation, and several psychosocial factors such as anxiety, depression, lower job satisfaction, and lower workplace social support at baseline.

**Conclusion:**

Over one-third of sonographers were at risk of developing a moderate-fluctuating disability trajectory. This unfavorable trajectory was associated with low physical activity level, poor ergonomics, psychosocial distress, and central sensitization at baseline.

**Impact:**

This study has important implications for the management of neck disability in workers. Addressing modifiable factors including low vigorous physical activity, poor ergonomics, anxiety, depression, and lack of workplace social support may improve the trajectory of work-related neck disability.

## Introduction

Neck pain and disability are important occupational health issues. Compared with employees such as nurses, physicians, and laboratory technicians who work in similar environments, sonographers who specialize in diagnostic examinations (sonography) using ultrasound are more likely to experience neck or other musculoskeletal pain.^1^ The prevalence of neck pain among sonographers ranges from 58% in Sweden to 95% in China,^2^^,^^3^ and 55% of sonographers experience at least mild disability in daily or work activities.^4^

The economic consequences of sick leave, reduced work productivity, and early retirement because of neck pain and disability have been well documented.^5–7^ A priority in the neck pain research agenda is to identify and define the natural or clinical course/trajectory of neck pain in general and specific populations.^8^ Understanding the trajectory patterns for neck disability in a working population with high risks of neck pain such as sonographers is essential for several reasons. First, it may enable workers with neck pain to be classified into clinically meaningful subgroups, facilitating more personalized physical therapy and workplace policy decisions. Secondly, it helps improve workers’ expectations regarding symptoms and prognosis, mitigates the potential negative influence of neck disability on their lives and assists with decision-making around self-management strategies. Moreover, it is important to recognize key characteristics and factors associated with diverse trajectories of neck disability to better direct patient-centered care.

Multiple factors have been found to be associated with neck disability, including individual, work-related, and psychosocial factors, and altered somatosensory function.^3^^,^^4^^,^^9^ However, these results were obtained from cross-sectional studies. There has been only 1 longitudinal study on sonographers, reporting that neck-shoulder pain at the 29-month follow-up was associated with pain intensity, adverse visual conditions, and high job demands at baseline.^10^ This study, however, was not designed to characterize the trajectory of changes in neck disability. Compared with single–time-point measurements, trajectories are considered more accurate measures of disability or pain status,^11^ and this type of analysis has been recommended to improve understanding of musculoskeletal pain.^12^ It remains unclear what patterns of neck disability trajectories sonographers would follow and which biopsychosocial factors influence or predict the observed trajectories.

Therefore, the 2 aims of this study were to identify and characterize sonographers with different trajectories of neck disability over 12 months and to investigate associations between observed trajectory patterns and biopsychosocial factors at baseline.

## Methods

### Study Design and Setting

This observational longitudinal study was conducted in Brisbane, Australia. At baseline from June 2018 to August 2019, participants attended 1 laboratory session to complete assessments of somatosensory function and questionnaires about individual characteristics, work-related (physical and psychosocial) factors, and general psychological factors. Participants received an online questionnaire to assess neck disability at baseline, 6, and 12 months. Up to 3 reminders were sent to participants within 2 weeks of each data collection time point; otherwise, their data for that time point were coded as missing, and data collection resumed at the next 6-month interval. Written informed consent was gained from each participant at baseline. This study was approved by The University of Queensland Human Research Ethics Committee (#2017001513) and complied with the Declaration of Helsinki. The STROBE guideline was used to prepare this report.

### Participants

Participants consisted of 92 sonographers working in Australia with and without neck disability at the baseline (n = 61 and n = 31, respectively). They were recruited through e-newsletters of relevant sonography associations, internal emails, and poster placements in private clinics and on social media. Participants were excluded if they performed sonography <4 h/wk; were pregnant; had a history of surgery in the spine or upper limbs or trauma; were diagnosed with systematic conditions such as fibromyalgia, irritable bowel syndrome, inflammatory conditions, or neurological disorders; or had undergone chemotherapy or radiotherapy for cancer within the past 5 years.

### Measurement of Neck Disability

Neck disability was assessed using the Neck Disability Index (NDI),^13^ which contains 10 questions concerning pain and activities of daily life. The NDI score was expressed as a percentage, with higher values indicating greater pain and disability.

### Baseline Assessment

Baseline biopsychosocial characteristics assessed included individual factors, work-related physical and psychosocial characteristics, general psychological factors, and somatosensory function as outlined below. These factors were selected based on our previous cross-sectional studies on risk factors^4^ and somatosensory features^9^ associated with neck disability in sonographers.

### Individual Characteristics

The individual characteristics assessed were age, sex, and time (hours per week) spent doing vigorous and moderate physical activities and/or walking. Characteristics were assessed using the International Physical Activity Questionnaire-Short Form.^14^

### Symptom-Related Characteristics

The 8 symptom-related characteristics studied were as follows: duration of neck pain (dichotomized as <3 months and ≥3 months on the basis of the definitions of acute pain and chronic pain^15^); sick leave because of neck pain in the past 3 months (no/yes); health care–seeking behavior because of neck pain in the past 3 months (no/yes); worst neck pain in the past week, measured on a 0- to 100-point numeric rating scale; baseline NDI score; eye complaints (eg, blurred vision, itching, dryness, and burning) (no/yes); interference of neck pain (no/yes); and total number of coexisting interfering symptoms for 5 regions, including shoulders, upper back, low back, elbows, and wrists/hands. Interfering symptoms were defined as symptoms that limited usual work or home activities.

### Work-Related Physical and Psychosocial Factors

Work-related physical factors included the number of working hours per week, physical demands as evaluated with a single question “How physically demanding is your job in a typical work day?” (anchored at 0 [nothing at all] and 10 [extremely heavy]),^16^ and the physical ergonomic risk as assessed with 6 items from the Job Requirements and Physical Demands Questionnaire.^17^ These items evaluated the time exposed to hazardous postures, including hands above chest level, forward arm reaching, backward arm reaching, head bending forward, awkward wrist postures, and applying pressure with the arm for ≥30 seconds at a time. Two additional items were included to assess exposure to neck twisting and shoulder abduction, which are reported as risk factors for musculoskeletal pain in sonographers.^18^ Total scores of ergonomic risks were computed by summing the 8 items. Higher scores indicated higher levels of ergonomic risk.

Work-related psychosocial factors were measured using the abbreviated version of the Job Content Questionnaire,^19^ with sum scores calculated separately for job control, social support, and psychological job demands. Scores were computed using the standard algorithm,^19^ with higher scores indicating better job control, higher psychological job demands, and more social support. Additionally, job satisfaction was evaluated by a single question (“How satisfied are you with your work?”) on a scale from 0 (totally dissatisfied) to 10 (highly satisfied).^20^

### Psychological Factors

The 7-item Generalized Anxiety Disorder Scale^21^ and the 8-item Patient Health Questionnaire^22^ were used to assess participants’ anxiety and depression levels, respectively. A total score of 5 or more represented mild anxiety or depression, which was defined as clinically relevant on both scales.^21^^,^^23^ The total scores and percentages of clinically relevant cases were computed and presented for a comprehensive understanding of depression and anxiety in sonographers.

The Pain Catastrophizing Scale was used to assess pain catastrophizing thinking.^24^ This scale has 3 subscales, including rumination, magnification, and helplessness. To better understand which specific catastrophic thinking sonographers may have, the total score and scores of each subscale were calculated, with higher scores representing more catastrophic thinking. Furthermore, participant’s beliefs about how work activities affect or would affect their neck pain were assessed using 2 items from the Fear-Avoidance Beliefs Questionnaire (“my work is too heavy for me” and “my work might harm my neck”).^25^ Total scores ranged from 0 to 12, with higher scores representing stronger fear-avoidance beliefs.

### Measures of Somatosensory Function

Quantitative sensory testing was conducted to assess somatosensory function, including the cold and pressure pain thresholds of a local site at the neck, and remote site at the tibialis anterior and the temporal summation of pain at the neck. Testing was performed unilaterally on the most painful site for participants with neck pain, or on the scanning hand side for asymptomatic sonographers. Measurement protocols for pain threshold and temporal summation of pain have been published elsewhere.^9^

### Data Management and Statistical Analysis

All statistical analyses were performed using R (version 3.4.2), and the R package LCMM^26^ was used to run the latent class growth analysis (LCGA, described below). Significance was set at *P* < .05.

### Missing Data

A total of 13 participants (14.1%) had missing data, with data missing for the NDI at 6 months (n = 5; 5.4%) and 12 months (n = 12; 13.0%) because of loss at follow-up. Compared with those who had complete data at all 3 time points, participants lost at the 6- or 12-month follow-up had significantly greater moderate physical activity, lower baseline NDI, lower number of coexisting interfering musculoskeletal symptoms, and higher work psychological demands (Suppl. Appendix). However, data were missing at random, as indicated by the Little Missing Completely at Random Test (*P* = .340). Therefore, missing data were handled by pairwise deletion, whereby information was discarded only when the particular data point required for a specific analysis was missing.

### Statistical Analyses

The LCGA was performed to identify classes of participants who followed similar trajectories of neck disability over 12 months. The LCGA was conducted because it enables identification of clinically relevant subgroups (trajectory classes) based on individual changes (eg, growth parameters) and further exploration of baseline predictive factors that characterize each of the subgroups. Briefly, observed repeated-measures data (NDI in this study) were used in LCGA to estimate grow parameters (eg, intercept, slope, and residual variance) of an individual participant. On the basis of maximum posterior probabilities, individual participants are then allocated to subgroups (latent classes) so that the growth patterns of individual participant’s disability trajectories are homogeneous within each class and heterogeneous between classes.^27^ The LCGA was conducted using the NDI value as a continuous dependent and time as a continuous independent variable as required for trajectory modeling.^27^ The LCGA model was built stepwise, with a pooled variance of intercept and slope, for 1 to 4 classes considering the modest number of participants. Then quadratic LCGA models were explored to allow possible nonlinear trajectories. To identify the optimal model, a combination of the following 3 criteria was applied^28^^,^^29^: model fit indexes, such as the Bayesian information criterion (BIC) and the Akaike information criterion (AIC), with lower values indicating better models and entropy with a value closer to 1 indicating greater homogeneity within each trajectory class; posterior class probabilities (probabilities with which each participant was allocated to each class), which were checked to assess the distinction between the classes, and number of participants per class; and interpretability of the identified classes regarding differences in growth parameters and clinical relevance. In the case that differences in the criteria between 2 models were small and possible interpretations of the trajectories were similar, the most parsimonious model was selected.^28^ A single outlier was identified during model building. This outlier had a very strong fluctuating disability trajectory (NDI = 82.0% for baseline, 36.0% for 6 months, and 68.0% for 12 months) compared with other participants. It was always placed in a class of its own, leading to difficulties in estimating meaningful models and preventing reliable statistical testing. Therefore, this outlier was excluded, and data from 91 participants were used for all statistical analyses.

Differences in baseline characteristics between the 2 trajectory class memberships (described in the Results section) were determined using independent *t* tests (normally distributed continuous variables), the Mann–Whitney *U* test (nonnormally distributed continuous variables), or the chi-square test (categorical variables). To describe how much each baseline factor contributes to the trajectory class membership, univariate logistic regression was conducted with each baseline factor as the independent variable and the trajectory class membership as the dependent variable, without covariates.

## Results

Of the 91 participants (83.5% women; median age = 37.0 [IQR = 31.0–48.0] years) included in the analyses, 72.5% were general sonographers and 27.5% were echocardiographers. They had 10.2 (IQR = 4.8–20.0) years of sonography experience and performed sonography for 27.5 (IQR = 20.0–34.0) h/wk.

### Trajectory Classes of Neck Disability

Overall, the linear and quadratic models had similar AIC and entropy values. However, the BIC values of all the linear models were smaller than the quadratic models. Therefore, based on the model selection criteria, linear models were preferred compared with quadratic models (Tab. 1). The growth indicators of linear models were presented in Table 2. Within the 4 linear models, BIC and AIC values decreased as the number of classes increased, but the differences were small. The entropy and posterior probability of the 2- and 4-class models were higher than the 1- and 3-class models, indicating that the former 2 models performed better (Tab. 1). Regarding the choice between 2- and 4-class models, 2 criteria that were particularly relevant were applied: the number of cases per class and the most parsimonious model was preferred when the differences between the criteria were small. In the 4-class model, the smallest class would have only 3 participants, and therefore the power to detect any differences in baseline characteristics would be lost. Furthermore, the differences in BIC, AIC, entropy, and posterior probability were small between the 2- and 4-class model; therefore, the more parsimonious model, which is the 2-class model, was preferred and used for further analysis.

**Table 1:** Model Fit Indexes for Latent Class Growth Analysis Models With 1 to 4 Classes*^a^*

**Models and No. of Classes**	**BIC**	**AIC**	**Entropy**	Minimum–Maximum Posterior Probability	**No. (%) of Participants/Class**
Linear models					
1	1850.4	1842.9	1.00	1.00	91 (100.0)
2	1799.7	1784.6	0.75	.92–.93	59 (64.8); 32 (35.2)
3	1799.3	1776.7	0.70	.82–.89	38 (41.8); 39 (42.9); 14 (15.4)
4	1795.8	1765.7	0.76	.83–.90	32 (35.2); 40 (44.0); 16 (17.6); 3 (3.3)
Quadratic models					
1	1853.1	1843.0	1.00	1.00	91 (100.0)
2	1804.7	1784.5	0.76	.89–.95	57 (62.6); 34 (37.4)
3	1806.6	1776.5	0.70	.84–.88	38 (41.8); 35 (38.5); 18 (19.8)
4	1802.2	1762.0	0.80	.86–.91	37 (40.7); 33 (36.3); 13 (14.3); 8 (8.8)

^a^ AIC = Akaike information criterion; BIC = Bayesian information criterion.

**Figure:**
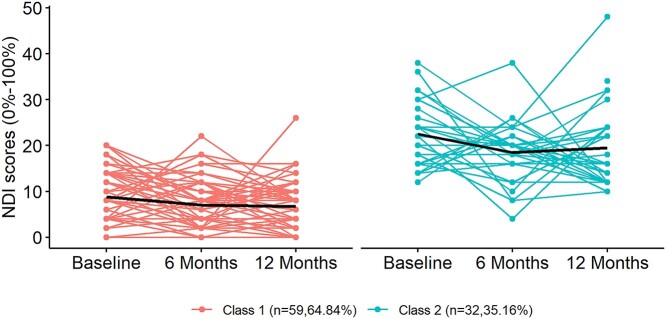
Identified trajectories of neck disability as measured with the Neck Disability Index (NDI). The *y*-axis represents NDI scores, and the *x*-axis represents the 3 time points over 12 months. The red and green lines represent individual trajectories within class 1 (low-resolving disability) and class 2 (moderate-fluctuating disability), respectively. The black line within each class represents the mean trajectories.

**Table 2:** Growth Indicators*^a^* Obtained in Latent Class Growth Analysis Models With 1 to 4 Classes

**Model**	**Parameters**	**Class 1**	**Class 2**	**Class 3**	**Class 4**
1 class	Intercept	13.10			
	Slope, mean (95% CI)	−0.19 (−0.41 to 0.03)			
	Error	8.75			
2 classes	Intercept	8.68	20.97		
	Slope, mean (95% CI)	−0.20 (−0.42 to 0.01)	−0.23 (−0.51 to 0.05)		
	Error	6.49			
3 classes	Intercept	6.22	15.90	24.38	
	Slope, mean (95% CI)	−0.19 (−0.45 to 0.07)	−0.30 (−0.60 to −0.01)	−0.13 (−0.92 to 0.66)	
	Error	5.88			
4 classes	Intercept	5.56	14.09	24.90	20.69
	Slope, mean (95% CI)	−0.19 (−0.44 to 0.07)	−0.22 (−0.46 to 0.02)	−0.62 (−1.09 to −0.16)	1.30 (0.32 to 2.29)
	Error	5.40			

^a^ Intercept, slope, and residual standard error of the model.

The 2 classes were labeled in a way that not only captured the subgroup mean but also the presentation of majorities of individuals in that subgroup, as recommended by a previous study.^12^ Class 1, representing 64.8% of the sample, was labeled as “low-resolving disability” because most participants in this class were characterized by a low level of initial disability with a slow linear decline toward no disability over 6 and 12 months (Fig.). Class 2, representing 35.2% of the sample, was labeled as “moderate-fluctuating disability” because most participants in this class showed a moderate level of disability at baseline with a fluctuating pattern over time (eg, a decrease in the NDI score at 6 months and then an increase at 12 months) (Fig.). Both classes included participants with relatively constant levels of NDI and participants with considerably fluctuating NDI scores.

### Relationship Between Baseline Factors and Trajectory Classes

The 2 trajectory classes had similar individual characteristics, except that the moderate-fluctuating disability class had lower vigorous physical activity levels compared with the low-resolving disability class (Tab. 3). The 2 classes differed in all symptom-related characteristics, except the presence of eye complaints, with odd ratios (ORs) ranging from 1.06 to 7.50 (Tab. 3).

**Table 3:** Baseline Characteristics and Odds of Being in the Moderate-Fluctuating Disability Compared With Low-Resolving Disability Trajectory*^a^*

**Variable**	Class 1: Low-Resolving Disability (n = 59)	**Class 2: Moderate-Fluctuating Disability** **(n = 32)**	**Odds Ratio** **(95% CI)**
Individual characteristics			
Age, y	36.0 (31.5, 45.5)	42.0 (31.0, 52.5)	1.02 (0.98 to1.07)
Women, no. (%)	47 (79.7)	29 (90.6)	2.47 (0.71 to 11.50)
Vigorous physical activity level, h/wk	1.5 (0.0, 3.8)	0.3 (0.0, 2.1)	**0.81 (0.63 to 0.99)**
Moderate physical activity level, h/wk	1.0 (0.0, 2.0)	1.0 (0.0, 2.1)	1.05 (0.89 to 1.25)
Walking time, h/wk	3.3 (1.5, 7.0)	3.5 (2.0, 10.9)	1.07 (0.99 to 1.17)
Symptom-related characteristics			
Neck pain duration ≥3 mo, no. (%) of participants^*b*^	39 (66.1)	29 (90.6)	**4.96 (1.52 to 22.46)**
Interference of neck pain, no. (%)^*c*^	15 (25.4)	23 (71.9)	**7.50 (2.94 to 20.63)**
Sick leave because of neck pain in past 3 mo, no. (%)^*d*^	7 (11.9)	13 (40.6)	**5.08 (1.81 to 15.39)**
Health care–seeking behavior in past 3 mo, no. (%)^*d*^	20 (33.9)	21 (65.6)	**3.72 (1.53 to 9.49)**
Worst pain in past 7 d, scored 0–100 on NRS	20.0 (2.5, 40.0)	60.0 (40.0, 70.0)	**1.06 (1.03 to 1.08)**
Baseline NDI, scored 0% to 100%	8.0 (4.0, 14.0)	22.0 (17.5, 26.5)	**1.53 (1.30 to 1.93)**
Eye complaints, no. (%)^*d*^	8 (13.6)	8 (25.0)	2.13 (0.70 to 6.45)
No. of body regions with coexisting interfering symptoms, scored 0–5	0.0 (0.0, 1.0)	1.0 (0.0, 2.0)	**1.63 (1.12 to 2.46)**
Work-related physical and psychosocial factors			
Work, h/wk	36.0 (24.0, 38.0)	38.0 (30.0, 38.0)	1.01 (0.96 to 1.01)
Physical demands, scored 0–10	6.0 (4.0, 7.0)	5.5 (4.8, 7.0)	1.00 (0.76 to 1.28)
Ergonomic risk, scored 8–32, mean (SD)	19.6 (3.4)	22.2 (3.4)	**1.25 (1.10 to 1.45)**
Job control, scored 24–96, mean (SD)	66.4 (8.0)	63.7 (10.5)	0.97 (0.92 to 1.01)
Workplace social support, scored 4–16, mean (SD)	12.5 (1.7)	11.1 (2.1)	**0.64 (0.46 to 0.83)**
Psychological demands, scored 3–12	7.0 (6.0, 8.0)	7.5 (6.0, 9.0)	1.21 (0.91 to 1.62)
Job satisfaction, scored 0–10	8.0 (7.0, 8.0)	7.0 (6.0, 7.0)	**0.46 (0.28 to 0.68)**
Psychological factors			
Depression: PHQ-8			
Total score, 0–24	2.0 (1.0, 4.0)	5.0 (3.0, 6.0)	**1.47 (1.22 to 1.85)**
Cases with PHQ-8 score ≥5,^*e*^ no. (%)	13 (22.0)	17 (53.1)	**4.01 (1.61 to 10.38)**
Anxiety: GAD-7			
Total score, 0–21	2.0 (0.0, 3.5)	4.0 (3.0, 7.0)	**1.34 (1.14 to 1.64)**
Cases with GAD-7 score ≥5,^*f*^ no. (%)	9 (15.3)	14 (43.8)	**4.32 (1.62 to 12.09)**
Pain Catastrophizing Scale			
Total score, 0–52	1.0 (0.0, 4.0)	3.5 (0.0, 11.0)	**1.11 (1.02 to 1.22)**
Rumination, scored 0–16	0.0 (0.0, 1.0)	0.5 (0.0, 3.0)	1.27 (1.00 to 1.62)
Magnification, scored 0–12	0.0 (0.0, 1.0)	1.0 (0.0, 3.0)	**1.43 (1.09 to 1.94)**
Helplessness, scored 0–24	0.0 (0.0, 1.0)	1.00 (0.0, 5.0)	**1.24 (1.05 to 1.51)**
Fear-avoidance beliefs, scored 0–12, mean (SD)	5.8 (2.9)	7.2 (2.3)	**1.23 (1.04 to 1.49)**
Somatosensory features			
CPT of the neck, °C	9.1 (6.3, 16.6)	10.2 (8.1, 14.9)	1.00 (0.94 to 1.07)
CPT of tibialis anterior, °C	6.5 (5.0, 11.7)	12.7 (8.4, 18.7)	**1.08 (1.02 to 1.16)**
PPT of neck, kPa	249.0 (201.3, 315.2)	210.8 (168.4, 261.4)	**0.994 (0.987 to 0.999)**
PPT of tibialis anterior, kPa	439.5 (354.7, 536.9)	334.6 (255.0, 463.3)	**0.996 (0.993 to 0.999)**
TSP at neck, scored as pain rating changes 0–100	8.3 (3.5, 16.7)	18.3 (6.7, 27.5)	**1.05 (1.01 to 1.09)**

^a^ Normally distributed continuous data are presented as mean (SD), nonnormally distributed data are presented as median (first quartile, third quartile), and categorical variables are presented as number (percentage). Odds ratios were obtained from univariate logistic regression. Bold type indicates that the variable was significantly associated with class 2. CPT = cold pain threshold; GAD-7 = 7-item Generalized Anxiety Disorder Scale; NDI = Neck Disability Index; NRS = numeric rating scale; PHQ-8 = 8-item Patient Health Questionnaire; PPT = pressure pain threshold; TSP = temporal summation of pain.

^b^ Reference: <3 mo.

^c^ Reference: no interference.

^d^ Reference: no.

^e^ Reference: PHQ-8 score <5.

*^f^* Reference: GAD-7 score <5.

Compared with the low-resolving disability class (class 1), the moderate-fluctuating disability (class 2) showed significantly lower workplace social support (*P* = .001) and job satisfaction (*P* < .001). For every unit increase of workplace social support and job satisfaction, the odds of developing moderate-fluctuating disability trajectory decreased by 36.0% and 54.0% (ORs = 0.64 and 0.46), respectively. In contrast, the moderate-fluctuating disability class had significantly higher ergonomic risk (*P* < .001), scores in depression (*P* < .001), and anxiety (*P* < .001), total score of pain catastrophizing (*P* = .039) and subscales of magnification (*P* = .026) and helplessness (*P* = .033), and fear-avoidance belief (*P* = .010) than the trajectory class of low-resolving disability. For every unit increase of these factors, the odds of developing moderate-fluctuating disability increased by a factor of 1.11 to 1.47 (Tab. 3).

Furthermore, all measures of somatosensory features, except cold pain threshold at the neck, significantly differed between 2 trajectory classes, with the trajectory class of moderate-fluctuating disability showing higher cold pain threshold (cold hyperalgesia) at the remote site, lower pressure pain threshold (mechanical hyperalgesia) at both local and remote sites, and higher temporal summation of pain (Tab. 3). The ORs of these somatosensory features ranged from 0.99 to 1.08 (Tab. 3).

## Discussion

This study identified 2 distinct trajectories of neck disability in sonographers, and they differed in some important variables such as vigorous physical activity, psychosocial status, and somatosensory function at baseline. These findings advance the knowledge of the natural course of neck disability over 12 months in health care workers, which will help facilitate individualized care.

### Trajectories of Neck Disability

The identified trajectories were labeled as low-resolving disability showing slow improvement toward no disability (64.8%) and moderate-fluctuating disability characterized by persistent moderate disability with a fluctuating pattern (35.2%). This finding indicates that neck disability in sonographers is heterogeneous regarding its course. Our results partly agree with Walton et al,^30^ who observed 3 different NDI trajectories over a month, with most neck pain patients (67.0%) showing a slow recovery from a mild baseline disability. Contrary to Walton et al,^30^ we did not identify trajectories of worsening and rapid improvement. The discrepancy may be because Walton et al^30^ investigated neck disability trajectories in a clinical sample of patients with either traumatic or nontraumatic neck pain who were undertaking physical therapy treatment for a month, whereas our study focused on the natural progression of neck disability in workers with nontraumatic neck pain over 1 year. Our results also partly contrast to Hallman et al,^31^ who revealed 6 trajectories of neck-shoulder pain intensity over a year in symptomatic and asymptomatic blue-collar workers and administrative staff. The 6 trajectories included maintaining asymptomatic (11.0%), constantly very low pain (10.0%), recovering from low initial pain (18.0%), improving from moderate initial pain (28.0%), presenting severe initial pain with a fluctuating pattern (24.0%), and persisting with very severe pain (9.0%). Several reasons may account for the differences between the current study and Hallman et al,^31^ including different sample sizes (91 vs 748), studied population (sonographers vs blue-collar workers and administrative, thus greater heterogeneity in work demands), frequency of data collection (3 vs 14 time points), and outcome measures (disability vs pain intensity).

Consistent with Hallman et al,^31^ this study did not identify a trajectory class with significantly worsening neck disability over time in the chosen 2-class model. However, in the 4-class model, 3 participants (3.3%) showed increased NDI scores over 12 months, with an average slope of 1.30 points/6 months. This 4-class model was not selected because with such a small number of participants, the power to detect any differences in baseline characteristics would be lost. One in 5 sonographers change their jobs or prematurely retire because of pain.^32^ This “healthy worker” effect^33^ may account for the lack of meaningful worsening trajectories, because those predisposed to worsening neck disability trajectories may have left the profession prior to the study or worked insufficient hours to be eligible for inclusion. Therefore, trajectories of neck disability may be different if stages of their working career are followed in the long term.

### Factors Associated With Trajectories of Neck Disability

As expected, the identified 2 trajectories of neck disability differed in various symptom-related characteristics at baseline, including baseline NDI, suggesting a clinical distinction between the trajectories. Compared with their counterparts, sonographers with chronic or interfering neck pain, sick leave, or health care–seeking behaviors at baseline had approximately 4 to 8 times (ORs = 3.72–7.50) higher odds of developing the moderate-fluctuating disability trajectory. These findings echo previous studies reporting that neck pain patients with worse clinical presentations were associated with poorer prognosis.^31^^,^^34^ Our findings suggest that information on various symptom-related characteristics could be useful in the early identification of sonographers at risk of an unfavorable outcome and help develop targeted management strategies.

There were clinically meaningful differences in vigorous physical activity at baseline between the low-resolving and moderate-fluctuating disability trajectories. The OR of vigorous physical activity was 0.81, meaning that for every 1-h/wk increase in vigorous physical activity, the odds of developing moderate-fluctuating disability trajectory decreased by 19.0%. This result corroborates previous findings that more time in vigorous leisure-time physical activity was correlated with a reduced likelihood of sick leave because of musculoskeletal pain among blue- and white-collar workers.^35^ Thus, improving vigorous physical activity levels may be an important intervention for reducing neck disability in workers such as sonographers.

Compared with the trajectory class of low-resolving disability, the class of moderate-fluctuating disability showed higher ergonomic risk, lower levels of workplace social support and job satisfaction at baseline. However, the difference was small and may not be clinically meaningful. On the other hand, univariate logistic regression analysis indicated that for every unit increment of social support (OR = 0.64) and job satisfaction (OR = 0.46), the odds of developing moderate-fluctuating disability trajectory decreased by 36.0% to 54.0%, indicating that workplace social support and job satisfaction could be important factors. Further research is needed to understand the relationship between these factors and neck pain to develop targeted intervention strategies.

The identified 2 trajectories differed in all general psychological factors at baseline, with the trajectory class of moderate-fluctuating disability showing poorer psychological status. This result confirms our previous findings with a cross-sectional design that sonographers with higher neck disability levels were associated with more impairments in psychological measures.^4^ When scores were dichotomized based on recommended thresholds for clinically relevant cases of depression and anxiety,^21^^,^^23^ 53.1% and 43.8% of sonographers in the trajectory class of moderate-fluctuating disability had depressive and anxious symptoms at baseline. Those showing depressive and anxious symptoms at baseline had approximately 4 times higher odds of developing moderate-fluctuating disability trajectory. In contrast, total scores of pain catastrophizing and subscales were very low across the 2 trajectory classes, and differences in the pain catastrophizing scale and fear-avoidance beliefs were small between classes, although statistically significant. These findings suggest that depression and anxiety may be important intervention targets to modify the progression of neck disability in sonographers.

Compared with the trajectory class of low-resolving disability, the class of moderate-fluctuating disability also demonstrated widespread hyperalgesia and heightened pain facilitation, suggesting that sonographers in this trajectory class were characterized by central sensitization (nociplastic pain mechanisms) at baseline.^36^^,^^37^ This finding lends support to our previous findings that hyperalgesia and heightened pain facilitation distinguished sonographers with moderate/severe disability from those with no disability.^9^ Previous longitudinal studies reported that altered pain sensitivity such as mechanical hyperalgesia and temporal summation of thermal pain predict worse outcomes of neck pain intensity and disability in the short term^38^ but not in the long term.^39^^,^^40^ These previous longitudinal studies, however, were designed to provide information on the prognostic value of pain sensitivity measures for neck pain and disability at 1 time point, such as 12 months. Therefore, they are not comparable with our findings. Our findings suggest that intervention strategies that target nociplastic pain mechanisms may improve outcomes in sonographers with neck disability. Individuals who followed the moderate-fluctuating disability trajectory had higher numbers of coexisting musculoskeletal pain sites, which suggests that a broader focus than the neck is needed.

### Limitations

Some limitations should be acknowledged. Firstly, there is no consensus on sample size calculation for LCGA, because it depends on the number of trajectory classes, heterogeneity between classes, and the relative proportions of participants per class. These parameters were not predictable at the stage of study design, and therefore it was not possible to calculate the required sample size. Because of the small number of participants in the trajectory class of moderate-fluctuating disability, univariate rather than multivariable logistic regression analysis was performed to test the association between baseline variables and the trajectory class memberships without considering confounding factors. This possibly introduced bias to the results. It is also unclear if having multiple risk factors at baseline increases the likelihood of developing moderate-fluctuating disability trajectory more than having 1 or 2 of the identified factors. Secondly, the sample consisted of a mix of participants with and without neck disability at baseline, which may confound the results. Finally, neck disability was only assessed at 3 points over 12 months. More frequently repeated measures may provide greater precision and accuracy of the trajectory of neck disability. Similarly, repeated measures of the baseline factors may provide important insights on the changes in the assessed biopsychosocial factors associated with fluctuations in neck disability. Therefore, future studies should preferably undertake more frequent repeated measures of neck disability and associated risk factors.

### Research and Clinical Implications

This study provides valuable implications for researchers and clinicians. The identified 1-year natural trajectories of neck disability could be used as comparators to assess the efficacy of future interventions for workers with neck pain. Although the population of interest for this study is sonographers, implications of our findings are expected to be generalizable to other occupations with similar physical and psychosocial work demands, such as dentists and laboratory technicians.

Individuals with neck pain often seek care from physical therapists. This study identified several symptom-related, psychosocial, and somatosensory characteristics that are predictive of an unfavorable disability trajectory. It is feasible for these characteristics to be identified using screening methods such as through history taking, questionnaire administration, and a clinical sensory test battery.^41^ The information provided should enable physical therapists to identify subgroups of workers such as sonographers at risk of developing unfavorable outcomes and tailor treatment or management strategies to individual workers. Considering that 64.8% of participants follow the trajectory of low-resolving disability, a “wait and see” approach or usual care may be an appropriate strategy for most workers, reserving more costly interventions for those with greater risk. Those who have identified indicators of the moderate-fluctuating disability trajectory most likely require management strategies at both an individual and organizational level, aimed at increasing vigorous physical activity levels, addressing altered somatosensory function, and improving psychological and work-related psychosocial status. On an individual level, pain neuroscience education^42^ and behavioral counseling^43^ may be critical, especially where central sensitization or pain beliefs interfere with their efforts to achieve more vigorous physical activity. Previous systematic reviews provide evidence that neuroscience education is helpful for coping with pain and improving psychological factors, such as pain catastrophizing,^42^ whereas behavioral counseling may help to increase self-efficacy related to physical activity among people with chronic painful musculoskeletal conditions.^43^ Combining these management techniques with physical therapy may be promising in addressing the modifiable risk factors identified. On an organization level, physical therapists are encouraged to work together with workers, occupational health and safety officers, and employers to improve work designs that help promote good physical and psychosocial work environment and vigorous physical activity at the workplace. Workplace-based physical activity of high intensity is believed to enhance musculoskeletal health and productivity if it is implemented by expert trainees such as physical therapists and supported by employers.^44^ However, the efficacy and cost effectiveness of these management strategies in shifting the unfavorable trajectory of neck disability into more favorable outcomes needs further investigation.

Two distinct trajectories of work-related neck disability were identified over 12 months: a “low-resolving disability” trajectory showing slow improvement toward no disability and a “moderate-fluctuating disability” trajectory characterized by persistent moderate disability with a fluctuating pattern. Over one-third of participants were at risk of developing a moderate-fluctuating disability trajectory. These participants, compared with those whose disability resolved over time, had lower vigorous physical activity, more severe symptoms, higher ergonomic risk, more psychosocial distress, and central sensitization at baseline. Our findings have important implications for the management of work-related neck disability in a high-risk occupation group.
